# Heat in Germany: Health risks and preventive measures

**DOI:** 10.25646/11651

**Published:** 2023-09-06

**Authors:** Claudia Winklmayr, Franziska Matthies-Wiesler, Stefan Muthers, Sebastian Buchien, Bernhard Kuch, Matthias an der Heiden, Hans-Guido Mücke

**Affiliations:** 1 Max Delbrück Center for Molecular Medicine in the Helmholtz Association (MDC) Berlin, Germany; 2 Helmholtz Munich Institute of Epidemiology Neuherberg, Germany; 3 German Alliance on Climate Change and Health (KLUG) Berlin, Germany; 4 German Meteorological Service Research Centre Human Biometeorology Freiburg, Germany; 5 Robert Koch Institute Department of Infectious Disease Epidemiology Berlin, Germany; 6 Stiftungskrankenhaus Nördlingen Department of Internal Medicine/Cardiology/Intensive Care Donau-Ries-Kliniken Nördlingen, Germany; 7 German Environment Agency Department of Environmental Hygiene Berlin, Germany

**Keywords:** HEAT EVENTS, MORBIDITY, MORTALITY, HEAT-HEALTH ACTION PLAN, VULNERABILITY, HEALTH PROTECTION, PREVENTION

## Abstract

**Background:**

Climate change has already led to a significant temperature increase in Germany. The average temperature in the past decade was approximately 2°C above the pre-industrial level and eight of the ten hottest summers since the beginning of systematic weather records in 1881 were recorded in the last 30 years.

**Methods:**

Based on a selective literature search and authors’ own results, the article summarises the current state of knowledge on heat and its health impacts for Germany, addresses adaptation measures, and gives an outlook on implementation and research questions.

**Results:**

Heat can aggravate pre-existing conditions such as diseases of the cardiovascular system, the respiratory tract, or the kidneys and trigger potentially harmful side effects for numerous medications. A significant increase in mortality is regularly observed during heat events. Previous approaches to mitigate the health impact of high temperatures include, for example, the heat alerts of the German Meteorological Service and recommendations for the preparation of heat-health action plans.

**Conclusions:**

Evidence on health impacts of heat and awareness of the need for heat-related health protection have grown in recent years, but there is still a need for further action and research.

## 1. Impact of heat on health in Germany

Rising temperatures and an increase in the frequency and intensity of heat events are among the most directly perceptible consequences of anthropogenic climate change. Other extreme weather events are discussed elsewhere in this progress report by Butsch et al. [1]. The health effects of high temperatures, including heat-related mortality, are increasingly becoming the focus of scientific and political initiatives, in Germany and internationally [2]. In the first part of this article we summarise the current state of knowledge on heat and its health impacts for Germany, in the main part we go into detail on adaptation measures that are being implemented and recommended, and in the third part we give an outlook on implementation and research questions that may arise. This compilation was completed at the end of May 2023 and reflects the status of the implementation of measures and recommendations at that time. As awareness of the health effects of heat and the need for countermeasures are constantly increasing, it is possible that further measures will have been decided upon by the time of publication and that individual recommendations have therefore already been followed.

### 1.1 Temperature changes in Germany

Climate change has led to temperature changes in Germany in recent decades. While the global mean temperature over land in the last decade (2011–2020) was 1.59°C (90% confidence interval (CI) 1.34–1.83°C) higher than the average temperature in the period from 1850 to 1900 [3], the temperature increase in Germany was 2.0°C (2011–2020 compared to 1881–1910, Figure 1a). The temperature increase has intensified significantly in the decades since 1990. Eight of the ten warmest summers since the beginning of systematic weather records in Germany (1881) were registered in the last 30 years.

**Figure 1:**
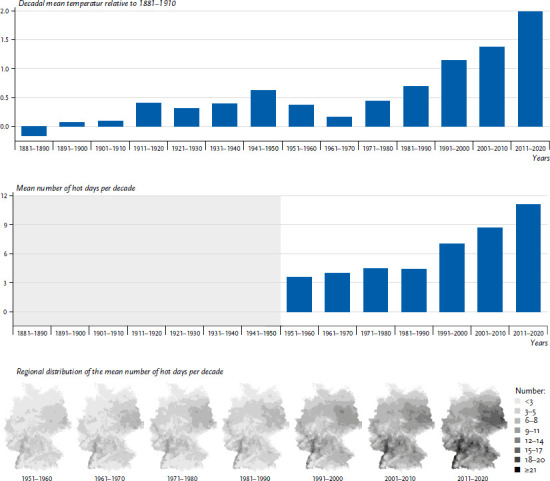
Source: German Meteorological Service (DWD) Figure 1a (top) Change in the decadal means of the annual mean temperature over Germany between 1881 and 2020. Shown are anomalies of the decadal means relative to the mean of the period 1881 to 1910. Figure 1b (middle) Area means of hot days in Germany since 1951. Shown are decadal means, no area means for the number of hot days are available before 1951. Figure 1c (bottom) Regional distribution of the number of hot days per decade since 1951. Days in legend are rounded down to next integer.

This development is also reflected in the number of hot days (days on which the maximum temperature reaches 30°C: Tmax≥30°C). This indicator can be evaluated with reliable accuracy across Germany from the mid-20^th^ century. Here, too, the strongest increase has occurred in the last three decades (Figure 1b). The increase in hot days affects almost all regions of Germany (Figure 1c). The highest absolute values occur in the south-west (Rhine Rift Valley and Rhine-Main region) and in the east (Berlin/southern Brandenburg). In the south-west, over 20 hot days per year were recorded regionally in the last decade, in the east, records show 16 hot days per year over a large area.

An identification of exceptional historical periods of heat, so-called heatwaves, is very much dependent on how a heatwave is defined and which region is considered. A uniform definition of the term heatwave does not yet exist (Info box 1). Since heatwaves are large-scale events, scientific analyses are generally not limited to Germany. An internal evaluation of the German Meteorological Service (Deutscher Wetterdienst, DWD) for the region of Central Europe (2–24°E and 45–55°N), in which heatwaves were defined as periods of at least three days during which the maximum temperature was above the 98^th^ percentile of a reference period, allows a ranking of heatwaves with regard to the affected area, the temperature anomalies that occurred and the duration. If the cumulative temperature anomalies are used as a measure of intensity, the heatwave of August 2003 ranks first among all heatwaves since 1950, followed by the heatwave of July/August 1994. The 2003 heatwave was also one with the longest duration to date, with up to 53 hot days recorded in the Upper Rhine area over the entire summer. The hottest heatwaves (in terms of cumulative temperature anomalies) occurred in 1994 and 2015.

Comparative studies across Europe also identify 1994 and 2003 as the years with the most pronounced heatwaves in Germany in terms of intensity and duration. In third place (depending on the criterion) are the years 2006, 2018, or 2019 [4, 5].

### 1.2 Future development according to different climate scenarios

As a result of climate change, the number of hot days and the duration and intensity of heat events will continue to increase in Germany. The extent of the increase depends, among other things, on the future development of greenhouse gas concentrations. With the help of regional climate models and under the assumption of concrete emission scenarios, it is possible to estimate the future development and the existing uncertainties for Germany (see also [10]).

**Info box 1 Heatwaves**

So far, there is no uniform definition of the term heatwave, neither in Germany nor internationally. Numerous proposals exist in the literature, which differ in the meteorological parameters used, the methodology for choosing threshold values and the criteria for minimum duration.

The possible parameters range from simple temperature variables (e.g. daily maximum air temperature) to combined human-biometeorological indices (e.g. perceived temperature [6]) to complex heatwave indices (heatwave magnitude index daily [4]). Sometimes the parameters are also subjected to temporal averaging (e.g. excess heat factor [7]). A combination of different parameters (e.g. daily maximum and daily minimum air temperature) is also used occasionally.

When choosing threshold values, a distinction can be made between absolute (e.g. daily maximum air temperature >30°C) and relative threshold values (e.g. based on percentiles) [8]. The minimum duration ranges from two days (e.g. [9]) to several days (e.g. five days [8]).

The wide range of possible heatwave definitions makes it difficult to compare the studies. However, the clear negative effect of heatwaves on human health can be found regardless of the precise heatwave definition. Throughout the article, we use the broader term ‘heat event’ to denote a prolonged period of severely elevated ambient temperature without further specified duration and intensity. Whenever we refer to a specific heatwave definition we will make the reference explicit.

**Figure 2:**
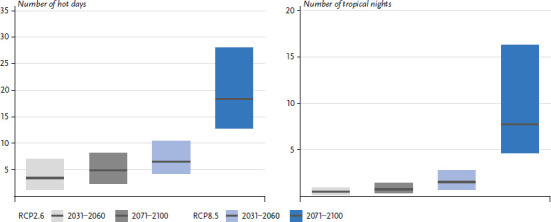
Projected change of hot days and tropical nights (mean across Germany; in days) compared to the reference period (1971–2000) for the near and distant future using the RCP2.6 and RCP8.5 scenarios. The ensemble median and the range are shown. Source: Brienen et al. [11]

Brienen et al. [11] have carried out such an estimation for the emission scenarios RCP2.6 (climate protection scenario), RCP4.5 (moderate scenario) and RCP8.5 (business-as-usual scenario) during the periods 2031 to 2060 (near future) and 2071 to 2100 (distant future), using an ensemble of regional climate models for different regions in Germany. Under RCP8.5, the temperature will rise by 3.0 to 4.2°C by the end of the century compared to the reference period 1971 to 2000, depending on region and season (ensemble median). Hot days will increase significantly in most regions as a result of climate change (Figure 2). In Germany, the average number of hot days under RCP8.5 will increase by about 18 days by the end of the 21^st^ century, with uncertainties between +13 and +28 days (Figure 2). Regionally, the increases vary, and in some regions the average number of hot days could rise to over 40 days per year. The coasts and regions of higher elevations also show significant increases.

In addition to the thermal stress during daytime, conditions at night also play a role during a heat event. Tropical nights, i.e. nights during which the air temperature does not fall below 20°C, are comparatively rare in Germany so far. Only very few, particularly warm stations record two or three tropical nights annually on average. In years with very hot summers, like 2003, however, more than ten tropical nights were observed at some stations. In Kehl near Strasbourg, for example, there were 21 tropical nights recorded in 2003. As a result of advancing climate change, tropical nights will occur more frequently in Germany. On average, Germany could experience up to 16 additional tropical nights per year by the end of the century (Figure 2), and in particularly warm regions even up to 30 additional nights.

**Figure 3:**
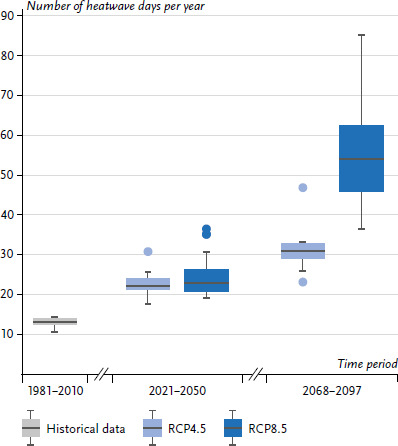
Mean number of heatwave days in the reference period 1981 to 2010 (left) and in the two emission scenarios RCP4.5 (moderate scenario) and RCP8.5 (business-as-usual scenario), each for the near and distant future. The boxplots show the interquartile range (IQR) as a box, the extent of the data as whiskers (lines with a maximum of 1.5*IQR) and outliers as points outside the whiskers. Source: Schlegel et al. [12]

Heat events will also continue to increase as a result of climate change, both in terms of duration, frequency, and intensity [12]. Defining heatwaves as periods of at least three days on which the daily mean air temperature is above the 95^th^ percentile of a reference period, Schlegel et al. [12] were able to calculate an increase in heatwave days of up to 22 to 23 days per year for the period 2021 to 2050 (RCP4.5 and RCP8.5) compared to 13 days in the reference period (1981–2010) (Figure 3). For the distant future (defined here as 2068 to 2097), there are greater differences between the emission scenarios. For RCP4.5, heatwave days increase to 31 days per year, for RCP8.5 there is an increase to 54 days per year. Compared to the reference period, this is a more than fourfold increase in heatwave days.

### 1.3 Heat-related mortality

**Figure 4:**
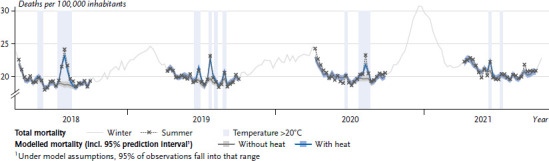
Example of modelling heat-related deaths. The slightly increased total mortality in spring 2020 and the strongly increased total mortality in winter 2020/2021 are due to the first and second wave of the COVID-19 pandemic. Source: Adapted from Winklmayr et al. [14]

Mortality statistics show the drastic effects of high temperatures on human health. A significant increase in overall mortality can regularly be observed during hot weeks (Figure 4). However, only for a very small proportion of deaths occurring during heat events high temperature is explicitly identified as the cause of death (e.g. between 7 and 60 deaths in the category ‘Damage due to heat and sunlight’ as classified by the causes-of-death-statistic by the German Federal Statistical Office [13]). Therefore, statistical methods must be used to quantify the relationship between heat and mortality.

#### Methods for estimating heat-related mortality

There are different approaches to quantifying heat-related mortality. First, there are methods that estimate the expected course of mortality without heat (background mortality): for this purpose, the average mortality of the previous years can be used [15, 16] or the seasonal course of mortality can be replicated using a periodic function, such as a sinusoid with an annual period [17, 18]. Both methods require a time series of mortality over several years and must ensure that excess mortalities that occurred in previous years do not distort the expected number of deaths. The number of heat-related deaths is then calculated as the difference between the observed mortality (during a heat event) and the expected background mortality.

**Figure 5:**
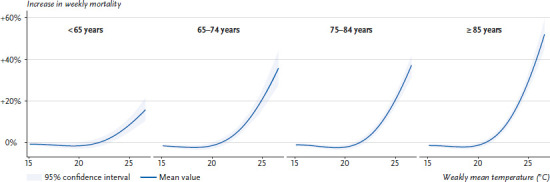
Exposure-response curves show the increase in weekly mortality as a function of the weekly mean temperature for four age groups (Example for greater region in Germany, period 2012–2021) Source: Own figure based on the modelling in Winklmayr et al. [14]

Another frequently used approach describes the influence of ambient temperature on total mortality by means of exposure-response curves [19, 20] (Figure 5). Such curves can quantify percentage changes in mortality as a function of temperature and allow us to investigate the systematic relationship between high outdoor temperatures and increased mortality. In particular, it is also possible to represent differences between geographical regions or demographic groups, or to analyse changes in exposure-response curves over time, which may provide evidence of successful adaptation. For example, the exposure-response curves shown in Figure 5 indicate that, on average, people aged 85 or over experience a significantly greater increase in mortality rate than people under 65 years of age for the same weekly mean temperature.

#### Heat-related mortality in Germany

A nationwide estimation of the heat-related mortality in Germany was first published in 2019 [21]. In the subsequent years, the methodology was further refined and the estimations were continued until the year 2022 [14, 22]. Additionally, some federal states also report regular heat-related mortality estimates [15, 23].

From 2018 to 2020, statistically significant numbers of heat-related deaths were estimated for the first time in three consecutive years. A total of 8,300 heat-related deaths were estimated for 2018, around 6,900 for 2019, and around 3,600 for 2020. In 2021, the number of heat-related deaths in Germany was not statistically significant. In 2022, the number of heat-related deaths was estimated at around 4,500 [22]. Although the increasing frequency of years with significant heat-related deaths is striking, heat-related mortality is not a completely new phenomenon: in the historically hot summers of 1994 and 2003, about 10,000 heat-related deaths per year were estimated, respectively [14].

The number of heat-related deaths in a population depends on a combination of several factors: the intensity and duration of heat events (heat exposure), the size of particularly vulnerable population groups, and also on how successfully adaptation measures have been implemented. The largest share of heat-related deaths occurs in the age group 75 years and older (about 75% of heat-related deaths). As a result of demographic change, a strong increase of the oldest population groups is to be expected in the near future [24], which may also increase the number of people potentially affected by heat-related mortality.

Due to their higher life expectancy, women make up about 60% of the age group 75 years and older, and are thus numerically more affected by heat-related mortality [23]. Per 100,000 inhabitants of the same age group, heat-related deaths occur to a comparable extent in women and men. However, the risk factors appear to differ between women and men [25].

The intensity of a heat event depends not only on the air temperature but can also be intensified by other meteorological parameters. For example, Muthers et al. [26] showed that mortality increased significantly more during the 2015 heatwave in Baden-Württemberg than during the 2003 heatwave, which can be explained by the significantly higher humidity in 2015. In addition, regional differences, for example with regard to building structures, healthcare, or age composition of the population, may also lead to differences in the number of heat-related deaths at comparable heat exposure.

Furthermore, in cities, the urban heat island effect also needs to be considered. On average, higher air temperatures can be observed near the ground in urban areas rather than in rural areas. The urban heat island effect is particularly important in the summer months and is more noticeable at night. The intensity of the urban heat island in a city depends, among other things, on the size of the city or the urban agglomeration, as well as on the building density. On clear summer evenings, maximum temperature differences of 10°C and higher have been observed between city centres and rural areas [27].

In the Berlin-Brandenburg region, a correlation between excess mortality during heat events and building density or the proportion of sealed surfaces, was found between 1994 and 2006 [28]. Increased soil sealing was accompanied by increased mortality, with the highest mortality rates being observed in the city centre. However, heat-related mortality is a significant problem in both urban and rural areas.

#### Heat-related mortality in other European countries

An increase of heat stress up to 1°C has been observed during the summer months of the last decades (1979–2016) [29]. Heat stress was determined using the universal thermal climate index (UTCI), a temperature equivalent, providing a measure of the average subjective human thermal sensation. It takes into consideration several meteorological variables relevant to the human thermal balance, such as air temperature, relative humidity, and wind speed, as well as the mean radiation temperature. A correlation with mortality data from 17 European countries showed that the relationship between the UTCI and the number of heat-related deaths depends on the thermal bioclimate of the observed country. Moreover, the study showed that the number of heat-related deaths increased with moderate (26–32°C UTCI) and severe heat stress (>32°C UTCI). In addition, mortality patterns may also differ depending on the thermal stress. In some countries, higher mortality rates appear to be associated to both high and low UTCI-values. In other countries, mortality increase was only observed under the influence of heat stress, while for some countries, a correlation between heat mortality and the UTCI could not be found [29].

### 1.4 Heat-related morbidity

The human body responds to heat stress with two key protective physiological mechanisms: (a) the redistribution of blood flow towards the skin (vasodilation) to remove heat from the muscles to the environment and (b) the secretion of sweat, which cools the body via evaporation [30]. These mechanisms in turn have an effect on other organs of the body: vasodilation requires increased contractility under the condition of reduced filling pressure. As a result, the heart has to beat stronger and faster, which requires a higher oxygen supply. In people with pre-existing heart conditions, this can lead to an imbalance between oxygen supply and demand, which can cause circulatory disorders (ischaemia), infarction, or circulatory collapse.

**Figure 6:**
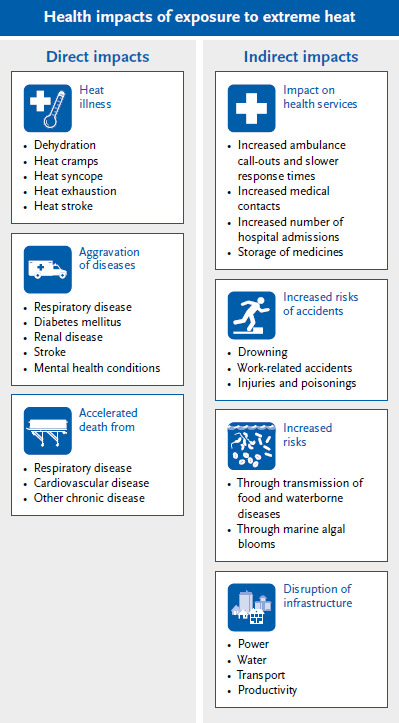
Direct and indirect effects of heat on health Source: Adapted from World Health Organization [35]

High temperatures and heat events can trigger heat-related illnesses as direct effects or aggravate pre-existing illnesses (heat-sensitive illnesses) and even lead to death (Figure 6). Mild and moderate heat-related illnesses include heat rash, heat oedema, heat-induced unconsciousness (heat syncope), heat cramps, and heat exhaustion, while heat stroke is a life-threatening consequence of heat [31, 32]. On hot days and during heat events, the risk of accidents increases, e.g. during manual labour or during leisure time (drowning while swimming). In addition, according to evaluations by the statutory health insurance, there is a demonstrable decrease in performance during heat events and an increase in heat-related sick leave [33].

The healthcare system is additionally burdened by hospital admissions and heat-related emergencies [30]. At the same time, healthcare structures can be affected by interruptions in their supply (e.g. power outages). Medical staff are also exposed to particular heat stress in the workplace, not least through protective clothing such as during the COVID-19 pandemic [34].

#### Cardiovascular diseases

In Germany, as in many other western countries, cardiovascular diseases are the most common cause of death. Based on data from 1993 to 2015 for major German cities, it was estimated that just under one percent of annual deaths from cardiovascular disease are attributable to heat [36]. In one study, it was calculated that the morbidity from cardiovascular diseases increases by 2.2% per 1°C increase in air temperature during a heat event [37].

Data from the Augsburg myocardial infarction registry show that severe temperature spikes can increase the risk of myocardial infarction [38]. Sun et al. [39] showed that there was an increase in hospital admissions with a diagnosis of myocardial infarction on days with high temperatures and on days immediately following these. Heat is also associated with increased rates of mortality from heart failure and stroke. People at high risk are the very old and those with pre-existing conditions (especially cardiovascular disease).

#### Respiratory diseases

Heat-related lung problems (e.g. pulmonary oedema, acute respiratory distress syndrome, increased pulmonary stress due to heat-induced hyperventilation and increased air pollution at high ambient temperatures) are the second most common cause of mortality and morbidity during heat events after cardiovascular diseases [30]. In addition to heat, traffic-related air pollution, especially in large cities, has an indirect effect on the morbidity and mortality risk for patients with chronic respiratory diseases [40] (see also the article in this status report on air pollutants by Breitner-Busch et al. [41]). In the summer months, on hot days in large cities, patients with chronic obstructive pulmonary disease (COPD) experience more frequent and sometimes life-threatening exacerbations. During heat, the body releases warmth not only through the skin, but also through the lungs, with a slight increase in respiratory frequency. However, this heat transport is restricted in people with pre-existing lung disease, so that efficient exhalation of heat is only possible to a limited extent. In addition, the muscle work required for the increased breathing movements in turn leads to an increase in body temperature. Finally, dehydration-induced reduced blood flow to the lungs makes the patient more susceptible to infections [42].

#### Metabolic diseases

People with diabetes are also at increased risk of being hospitalised during heat events. Similar to lung disease, these patients have impaired thermoregulatory responsiveness in the lungs at the vascular level, as heat exposure affects the self-regulation of blood vessels, leading to an increased tendency to form clots [43].

#### Kidney disease

Reduced blood volume due to dehydration can cause damage to the kidneys. As has been observed, especially in hot regions of the world, in people who work outdoors, chronic dehydration can cause renal fibrosis (scarring of the kidney) and chronic kidney disease [30].

#### Effects on pregnant women

There is increasing evidence that high temperatures can affect pregnant women and the course of pregnancy, e.g. through reduced blood supply via the placenta, dehydration, or inflammatory processes that can trigger pre-term birth [44, 45]. For example, a meta-analysis estimated that the probability of pre-term birth is 1.05 (95% confidence interval (CI): 1.03–1.07) times higher for every 10°C increase in temperature and 1.16 (95% CI: 1.10–1.23) times higher during heatwaves (defined as two or more days with temperatures above the 90^th^ percentile). Heat exposure of pregnant women over the course of pregnancy can also lead to low birth weight (<2,500 grams) of the newborn [46].

#### Interactions with medication

In addition to the factors mentioned above, the intake of medication also plays an essential role in the effects of heat on the individual [47, 48]. Table 1 lists a number of medications and groups of active substances that can have dangerous side effects during heat (Table 1 supplements the table in [48]).

Heat-induced vasodilation can significantly increase the antihypertensive effect of many cardiovascular drugs, with the consequence of syncope with possible serious injury (for example due to falls), critical organ ischaemia (organ circulatory disorders), or even myocardial infarction. People with systolic heart failure (cardiac insufficiency) and patients with high blood pressure are particularly at risk. In hot temperatures, antihypertensive drugs, which are often prescribed to treat cardiac insufficiency, can lower blood pressure too much. The combination with diuretic drugs is particularly problematic: these are used (alone or in combination with other antihypertensives) to lower blood pressure, but also for drainage in people with cardiac insufficiency [48].

Drugs that inhibit the effect of the neurotransmitter acetylcholine (anticholinergics) can also lead to complications during heat, as they influence central temperature regulation and prevent sweating. This anticholinergic effect is contained in many medicines that are used for a wide range of complaints (bronchial asthma to urinary incontinence) [49].

Other large groups of medications that can develop adverse effects in heat include agents used to treat cardiac arrhythmias (antiarrhythmics), agents used to treat coronary artery disease (antianginosa), and antidiabetics. Various painkillers also carry a high interaction potential, especially opiates that are administered for absorption through the skin [50].

Even in younger persons, who suffer from pre-existing conditions less frequently, certain medicines show potentially dangerous side effects when exposed to heat. For example, an analysis of the Augsburg MONICA/KORA myocardial infarction registry recently showed that patients taking antiplatelet drugs or beta-blockers are more vulnerable to non-fatal myocardial infarctions due to heat exposure than people not taking these drugs [51].

The comparatively new group of active substances called SGLT2 inhibitors, which are used in the treatment of diabetes patients, can also lead to undesirable effects in heat. In this case, there is an increased excretion of glucose and thus volume via the urine. This group of drugs is important to observe, because it will be used in a large number of patients due to its significant expansion of indications (in addition to diabetes all forms of heart failure and prospectively also renal failure) [52, 53].

Furthermore, the shelf life of medicinal products is generally impaired by heat, which can reduce their effectiveness [54]. Many medicines have an extensive interaction potential, which can be massively increased by the effects of heat.

### 1.5 Vulnerable population groups

Population groups that are vulnerable to heat-related morbidity and mortality due to their risk factors include older persons (especially those living alone), people with pre-existing conditions (especially cardiovascular disease, respiratory disease, kidney disease, obesity, diabetes), pregnant women, infants and young children, people who do manual labour outdoors or engage in intensive sports, people with physical and mental impairments, people with low socioeconomic status, and the homeless [31]. The issue of health equity in climate change is also addressed in an article by Bolte et al. [57] in this status report.

In order to avoid as much heat-related illness and death as possible, heat-health action plans (HHAPs, discussed in detail in Section 2.2 Heat-health action plans for health protection), and other measures should primarily target those who are at increased health risk during heat events. Table 2 provides an overview of the affected population groups.

## 2. Adaptation measures for heat-health protection

### 2.1 The heat-health warning system of the German Meteorological Service (DWD)

To implement adaptation measures, it is first necessary to predict upcoming heat events and to swiftly inform the population and the healthcare system. In Germany, this task is performed by the heat-health warning system of the DWD [59], which was put into operation nationwide in the summer of 2005, in response to the numerous additional deaths throughout Europe (e.g. [60]) in the summer of 2003.

A physiologically relevant assessment of heat events must take into account not only the air temperature but also the water content of the air (humidity), as well as the wind and radiation conditions. In the DWD heat-health warning system, this is done using the perceived temperature [6]. The perceived temperature describes the temperature perception of a ‘reference person’ and translates the current thermal conditions into the air temperature of a standard environment. For the perceived temperature, there are categories of heat stress that enable a thermally relevant evaluation of the current conditions [6].

Heat alerts are issued by the DWD at the municipal level for the current and following day. The alerts consider the respective altitudes, so that an alert may only be valid for parts of the municipality (e.g. areas below 800m altitude). Two warning levels are distinguished for heat warnings: a ‘strong heat stress warning’ is issued when the perceived temperature exceeds a threshold of 32±2°C; an ‘extreme heat stress warning’, on the other hand, is issued when the perceived temperature is expected to exceed the threshold of 38°C in the early afternoon (2pm). The threshold for severe heat stress varies over the course of the summer depending on the meteorological history. If the preceding weeks were cooler (warmer), the threshold is somewhat lower (higher) [18]. This approach accounts for the short-term adaptation to heat that takes place over the course of each summer.

In addition to the conditions during the day, warnings of strong heat stress also take the conditions during the night under consideration. Since most people stay indoors at night, a building simulation model is used to determine the indoor temperature of a standard building [61]. This temperature must be above certain, regionally adapted threshold values before a warning of strong heat stress can be issued. The warnings are supplemented by additional modules that deal more precisely with the burden on older persons and people in need of care, and take into account the effect of the urban heat island.

In Germany, current heat alerts are distributed via e-mail newsletter, the DWD alert page or various smartphone apps. In addition, heat alerts are disseminated via civil protection warning systems. The e-mail newsletter also includes situation-specific behavioural recommendations. Nursing homes and other healthcare providers are encouraged by the state health offices to subscribe to the DWD newsletter.

Once a heat alert has been issued, the health authorities of the federal states are called upon to initiate intervention measures. In addition to the heat alerts for the next 48 hours, the DWD also provides advance heat information for the next two to eight days [62].

### 2.2 Heat-health action plans for health protection

Until the extremely hot summer of 2003 with its catastrophic health effects across Western Europe, most European countries lacked appropriate precautionary planning to protect the population from the health effects of extreme heat. Based on initial studies, findings, and experiences, the World Health Organization’s (WHO) Regional Office for Europe published guidelines for heat-health action plans (HHAPs) for health protection in 2008 [63]. HHAPs serve as an instrument for establishing targeted measures for preventive health protection, and have since been established in several European countries, such as France, Italy, and Switzerland.

**Figure 7:**
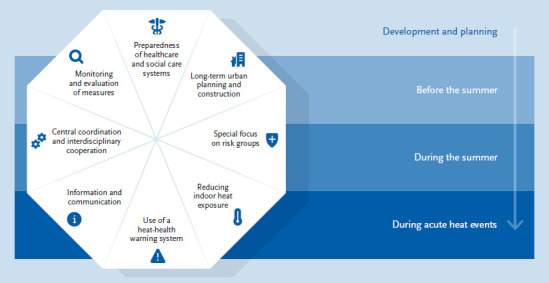
The eight core elements of a heat-health action plan with their temporal implementation: most core elements are implemented in each preparation and protection phase Source: Adapted from World Health Organization [63] and Straff et al. [64]

The overall goal is to reduce the health risk of extreme heat events and to strengthen individual resilience to adverse developments. In order to be better prepared for the environmental changes caused by climate change (some of which have already occurred) targeted adaptation measures are necessary in addition to climate protection. In 2017 in Germany, a working group comprised of federal and state actors, commissioned by the Federal Ministries for the Environment and of Health, drew up recommendations for the development of HHAPs for the protection of human health on the basis of the WHO guidelines [63], which should enable federal states and municipalities to develop concrete plans of action and put them into implementation [64]. They outline short-, medium-, and long-term options for health adaptation measures for a timely and gradual implementation of regionally adapted HHAPs. These recommendations are based on eight core elements and set a framework for the development of individual immediate measures, which are to be implemented during an acute heat event, preparatory, preventive and treatment measures in healthcare and nursing facilities with special consideration of vulnerable population groups, and adjustments in building and urban/spatial planning, which can only yield results in the longer term (Figure 7).

While the federal level provides the framework for climate change adaptation, as with the ‘German Strategy for Adaptation to Climate Change’ [65] and the HHAP recommendations [64], the responsibility for implementing concrete adaptation measures lies predominantly at the municipal level [66]. However, as extreme heat events and their consequences do not occur evenly across Germany, regionally and locally specific conditions must be considered. Therefore, adaptation measures follow the subsidiarity principle, i.e. concrete measures should be implemented competently and responsibly at the most appropriate level. The implementation of HHAPs at the municipal level is strongly recommended [34, 67], but is not yet legally required. Adaptation to the consequences of climate change, including heat, is supported by the federal government through various funding initiatives, which municipalities can apply for to support the implementation of relevant projects.

Several studies in recent years have investigated whether and how the HHAP recommendations have been taken up by federal states and municipalities and which plans and adaptation measures to prevent heat-related health impacts have already been implemented [68–71].

The results of an online survey conducted in Germany in spring of 2020 show that the health relevance of heat is recognised as an interdisciplinary issue at the federal, state, and municipal levels and is addressed by both the environment and health agencies at each level [70, 72]. In some municipalities and recently also at the state level, HHAPs are being implemented [15, 70, 73]. The federal states and municipalities are particularly active in communicating information and educational materials, thus contributing to behavioural prevention by allowing individuals to protect themselves against extreme heat, often through low-threshold, easy-to-implement adaptation measures. For example, many cities list tips for their citizens on their websites or in printed guidelines on how to correctly behave in hot weather. Overall, the number of heat prevention measures and projects in Germany has been steadily increasing [74] since the introduction, dissemination, and promotion of the HHAP recommendations in 2017 [64], and HHAPs have recently been developed at the level of municipalities and federal states (Section 2.4 Positive examples of heat-related health protection). However, it remains unclear whether this fulfils the criteria for effective short- and medium-term protection, as individual projects only include some of the eight core elements of the recommendations [68].

### 2.3 Further recommendations for heat prevention and heat-related health protection

The evidence for effective heat-related health protection is well known among decision makers and a wide range of information materials is available. Recently, the number of concrete interventions and action plans in the health and nursing sectors has been increasing, and the range of educational modules on the topic has been growing steadily for several years. This is shown by a working document related to the development and implementation of a HHAP for cities and municipalities [75]. A collection of concrete implementation examples from municipal practice is provided in Info box 2 [75]. Some information materials and recommendations for measures in the health sector were adapted to consider the COVID-19 pandemic (e.g. [34, 76]).

In summer 2021, key players from the German health sector were surveyed in semi-structured interviews on the implementation of integrated HHAPs in order to review the recommendations of the 2019 Lancet Countdown on Health and Climate Change Policy Brief for Germany [67]. In line with nationwide studies [68, 70, 75], the experts estimated that so far only a few municipalities have implemented comprehensive and integrated HHAPs in which stakeholders from the health sector, such as the medical and nursing professions, emergency services, and clinics, have been successfully involved in the development of the plans. It is crucial for authorities and actors in the health sector to consider heat-related health protection their responsibility in order to develop initiative and active participation. Kaiser et al. [70] show, however, that the mere existence and awareness of the recommendations is not sufficient for municipalities to successfully develop HHAP. For example, some municipalities lack the financial and human resources to develop and implement HHAPs. The lack of a legal basis enabling binding action with planned resources is also pointed out [70].

**Info box 2 Selection of proposals and recommendations for heat protection for use in the health sector**

Health effects due to heat and periods of extreme heat can largely be avoided. Prevention requires a range of measures at different levels: from public health precautions coordinated with meteorological early warning systems, to timely official and medical advice, to improvements in housing and urban planning. The following selected current materials (some in German) serve this purpose:

► WHO public health advice on preventing health effects of heat – new and updated information for different audiences (2011)
► Climate change and education. Different educational modules for various professional groups (including paediatricians, medical assistants and nursing staff, youth work) in the health sector provided by the Ludwig-Maximilians-Universität München (2020–2022)
► Heat as a field of action at the German Alliance on Climate Change and Health (2022)
► Climate-Human-Health. Website of the Federal Centre for Health Education on heat and heat protection (2022)
► Climate change and health: Tips on coping with hot weather and heatwaves. Advisory brochure of German Meteorological Service and German Environment Agency (2019)
► The heat etiquette booklet: Tips on how to act in hot weather. Brochure of the German Environment Agency on heat protection (2021)
► Working aid for the development and implementation of a heat-health action plan for municipalities from Fulda University of Applied Sciences (2023)
► Governmental recommendations for action for the preparation of heat action plans to protect human health (2017)

This compilation is a selection and makes no claim to completeness.

In June 2022, the conference of German federal health ministers, in cooperation with federal states’ working groups, decided on an implementation concept to accompany the decision from 2020 [77]. In the meantime, the first federal states, such as Hesse and North Rhine-Westphalia, have issued declarations or resolutions for the implementation of HHAPs. In autumn 2021, the 125^th^ German Medical Assembly also passed several resolutions under the slogan ‘climate protection is health protection’ [78], to reduce the high proportion of between 5 and 6.7% of Germany’s overall greenhouse gas emissions caused by the health sector and to move towards climate neutrality [41, 79].

### 2.4 Positive examples of heat-related health protection

There are a number of positive examples of heat-related health protection in Germany that have already been implemented in practice. They range from the development of information materials for vulnerable population groups and institutions to HHAPs and alliances for action in the health sector. Training and qualification tools on heat and health, such as the educational materials of the Ludwig-Maximilians-Universität Munich (LMU) or the German Alliance on Cllimate Change and Health (KLUG), are also available. In the following, a selection will be presented.

#### First heat-health action plans

Since their publication in 2017, the HHAP recommendations [64] have been continuously and widely disseminated through various communication channels, presented at different events to expert audiences from science and public administration as well as to a broader expert public and stakeholders (e.g. at national symposia, conferences, and further education events of the public health service) and published in various journals. In addition, in 2021/2022, some municipalities and federal states received initial consulting advice for concrete HHAP implementation from Fulda University of Applied Sciences, from state institutions such as the Centre for Health of North-Rhine Westphalia, from the Centre for Climate Adaptation serving for the German Adaptation Strategy on Climate Change and other research, advisory, and non-governmental organisations.

The 2020 online survey described above showed that the respondents consider the HHAP recommendations a predominantly positive helpful basis for their work and an easily understandable and practicable guideline for a HHAP. In some federal states and municipalities, a number of impulses and developments have already been triggered and some health-related adaptation measures for heat prevention have been initiated [70]. Since then, the first concepts for HHAPs at the municipal level have been developed on the basis of these recommendations, as compiled in Info box 3.

#### The Swiss-style heat toolbox

Based on the original template from Switzerland from 2017 [80], the Bavarian Health and Food Safety Authority developed the Bavarian ‘heat toolbox’ [81]. On coherently presented fact sheets, it provides support for the design, planning, and preparation of HHAPs. In addition, examples of successful implementation are presented [81]. The experiences of heat-health action planning from Switzerland show that successful prevention is based on a variety of measures (before, during, and after the summer) and strengthens individual health competence, thus promoting health.

**Info box 3 Selection of first heat-health action plans**

Since the publication of the governmental recommendations for action for the preparation of heat-health action plans (HHAPs) in 2017, first HHAPs had been developed at the municipal level by the end of 2022, mainly supported by various federal funding initiatives. They include (in German):

► Offenbach am Main 2020
► Mannheim 2021
► Cologne 2022: HHAPs specifically for the elderly
► Worms 2022
► Federal state of Brandenburg 2022: Expert review of a HHAP
► Federal state of Berlin 2022: Aktionsbündnis Hitzeschutz Berlin (first model HHAP for outpatient and inpatient care, medical practices and the public health service)
► Federal state of Hesse 2023: Hessian HHAP

Especially in the warmer regions of western Switzerland, a reduction in heat-related mortality risk has been observed in recent years. There are indications that the cantonal HHAPs and thus the coordinated activities for the prevention of negative heat effects on the population have contributed to this reduction. In general, the targeted education of the population about heat-health risks appears to be important. Various projects investigating the effects of heat on health in Switzerland have led to an improvement in the knowledge and action basis in recent years. The presentation and dissemination of the results at stakeholder meetings and to the general public via the media has contributed significantly to raising awareness of this issue [80].

First evaluations report successes for regions with HHAPs compared to those with isolated measures [80]. This corresponds to international reports, although only a few studies on HHAP evaluation are available so far [82]. In Rome, Italy, there are reports of successes of the ‘Long Live the Elderly’-programme [68], which is based on the involvement of neighbours as volunteers, the so-called buddy system. This has yet to be developed for Germany.

#### Experience from the care sector

As a reaction to heat-related deaths in the summer of 2003, a heat-health warning system and a set of measures for care facilities were developed in Hesse to protect vulnerable persons during heat events, driven by an interdisciplinary group of decision-makers and actors involved in care [83]. The practical recommendations, which were first published in 2009 and updated in 2017, cover topics ranging from the identification of residents who are particularly at risk to heat-related health protection measures in care and nursing. Options for regulating room temperature and indoor climate are also presented. Since 2004, the Hessian care and nursing administration has been inspecting and advising inpatient facilities on heat alert days regarding immediate measures, legitimised by a decree of the Hessian Ministry for Social Affairs, with the results being documented. It has been reported that heat-related health protection has become established in the care and nursing facilities in Hesse since the introduction of this system [83].

#### An alliance for heat-related health protection in Berlin

**Figure 8:**
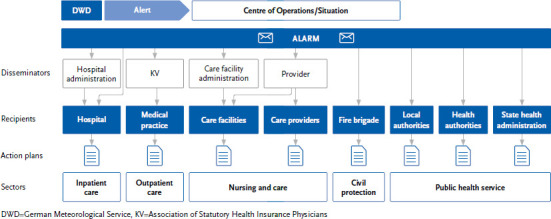
Established heat alert cascade in the pilot phase of the ‘Aktionsbündnis Hitzeschutz Berlin’ Source: Barker [84]

The pilot project ‘Aktionsbündnis Hitzeschutz Berlin’ (Action Alliance for Heat-Related Health Protection Berlin), which was launched in March 2022, prioritises those heat protection measures in the health and care sector that increase the protection of vulnerable population groups during an acute heat event [73]. This formation of an essential, interdisciplinary network of actors for heat-related prevention and adaptation in the medical sector at its core was achieved through the responsibility, insight, and initiative of healthcare actors. The network developed a heat alert cascade for the healthcare sector (Figure 8) and concrete interventions, recorded in sector-specific action plans [73, 84]. The ‘Aktionsbündnis Hitzeschutz Berlin’ and its experience can serve as a helpful example for other cities, municipalities, and federal states, specifically the model heat protection plans for various facilities, which were designed as low-threshold checklists.

## 3. Outlook: Need for action and research

Current climate projections suggest that the probability of heat events in Central Europe will more than double by the end of the century and that they could last 30 percent longer. As a result, heat stress in an ageing German society is expected to increase and therefore heat-related illnesses and mortality rates could continue to rise if no effective counter-measures are taken [85].

An increase in frequency and intensity of heat events could lead to healthcare facilities being temporarily unable to fulfil their function because of overburdening or because of infrastructure failures. It is undisputed that climate change and the growing challenges posed by extreme heat are likely to cause considerable additional costs for the healthcare sector in the medium term, on the one hand due to the care of patients, but also due to awareness campaigns, the adaptation of infrastructure, and the training of staff. In the long term, however, the costs for the healthcare system could again decrease, provided that preventive measures are successfully implemented [85].

Measures of structural prevention for adapting to high temperatures and heat events such as the creation of green spaces and parks, on the one hand benefit the local or urban climate, and on the other hand contribute to the health of the population, e.g. by improving air quality and by providing recreational spaces. Furthermore, climate-friendly individual behaviour (behavioural prevention) usually also results in substantial health benefits, so-called health co-benefits, in addition to climate protection (see also [10]). Recommendations for behavioural prevention that are also climate-friendly thus contain health promotion potential. The open-mindedness and the agreement on the necessity of HHAPs has clearly grown in recent times among health professionals, associations in the health and care sector, in the public health service, and the municipalities.

### 3.1 Recommendations

There is still a need to improve heat-related health protection and heat prevention in Germany. This requires (a) addressing the overarching structural deficit of the public health service, (b) systematic and nationwide implementation of HHAPs that integrate acute and medium- to long-term measures relating to the eight core elements, and (c) anchoring heat prevention and heat-related health protection in the health sector and among the general public. Specific recommendations on measures in the fields of action that contribute to these three goals (structural prevention, health promotion/healthcare/health protection, civil protection, education and training, health awareness and communication, surveillance, urban and spatial planning, and occupational health and safety) are summarised in Table 3. They range from setting policy frameworks for the implementation of HHAPs to the active involvement of healthcare actors and the review of building and occupational health and safety legislation.

Implementation and accompanying research on concrete projects help determine which factors promote the implementation of HHAPs and heat-related health protection measures in relevant institutions, municipalities, districts, and federal states, and which make it more difficult. The results can be used to tailor the implementation process in new projects. Continuous monitoring and evaluation of HHAPs (both of the process and the effectiveness) are needed to determine the effectiveness of HHAPs and integrated measures (Table 3). This can provide information on which factors influence the population’s capacity and ability to adapt to high temperatures and heat, and which measures are helpful in supporting the population to adapt and make them more resilient.

### 3.2 Research needed in medicine, epidemiology, and health services research

Even though evidence regarding adverse health effects of high temperatures has increased in recent years, there are still a number of open research questions on the various relevant topics and fields of action. In accordance with the research questions listed here and those that will arise in the future, it is important that funding programmes of the respective authorities are appropriately equipped and directed.

#### Medical/epidemiological issues

Above all, more detailed knowledge is required on the effects of high temperatures or heat on morbidity: there are open questions about the role of pre-existing conditions (e.g. cardiac insufficiency, heart attacks, strokes, diabetes, kidney disease, COPD, asthma) and an estimate of the number of people affected, e.g. by calculating the number of years of life lost (YLLs) and the number of years lived with disability or disease (YLDs). Possible long-term effects of high temperatures and heat have hardly been studied so far. The question of how exactly pregnancy complications relate to temperature, and through which pathophysiological mechanisms, has also not yet been answered in detail. Interactions in the combined use of different heat-sensitive drugs require further investigation, along with the development of appropriate preventive measures. The question of the impact of additional environmental factors that strongly influence the effects of heat events on morbidity and mortality (interactions and synergies) should also be further investigated.

#### Health services research

According to the Climate Impact and Risk Assessment for Germany 2021 [85], only a few studies examine the consequences of climate change on the healthcare system or the health economy – there is a need for further research in this area.

In the context of health services research, the question arises as to what role heat-related deaths play in hospitals, in care facilities, and in private residences [85]. In order to prepare health services for the effects of heat events, it is important to investigate how many additional patients attend doctors' offices and emergency rooms during heat events and what costs are associated with this. More precise knowledge about the extent to which employees in health and care facilities are affected in their work in high temperatures (especially indoors) is necessary to protect them and to ensure necessary medical care and nursing during heat events.

## 4. Conclusion

As climate change progresses, a further increase with respect to the frequency, duration, and intensity of heat events is to be expected in Germany. The population, the healthcare system, and the municipalities are not currently sufficiently prepared for particularly extreme events. Heat events must be taken seriously as an increasing health risk and the federal, state, and local governments, as well as the health and care institutions, have a duty to strengthen existing measures or take new steps to minimise risks, taking into account new findings, and to support the development of resilient structures [84]. The healthcare sector should play a central role in this.

## Key statements

- As a result of climate change, temperatures in Germany are increasing more strongly than the global average.
- During heat events, a significant increase in mortality is regularly observed, especially in older age groups.
- High temperatures and heat events can trigger heat-related illnesses, aggravate pre-existing conditions and even lead to death.
- Various medicines, especially antihypertensive drugs or diuretics, can have dangerous side effects during heat.
- Heat-health action plans serve as an instrument for establishing targeted intervention measures for preventive health protection and health promotion.
- The healthcare sector should play a key role in the development and implementation of heat-health action plans.
- Heat-related health protection must always be considered in the context of climate protection. Ultimately, climate protection leads to sustainable health protection.

## Figures and Tables

**Table 1:** Drug interaction/side effect potential during heat events, grouped by active substance/indication class Source: Adapted and expanded based on Kuch [48]

Active substance/indication class	Examples	Type of adverse reaction aggravated by heat
Anticholinergic medicines (these include many psychotropic drugs, antidepressants, but also bronchoinhalatives, etc.)	Many benzodiazepines (sleeping pills), promethazine (antipsychotic), tricyclic antidepressants, ipra-/tiotropium bromide (COPD), trospium chloride (for incontinence), biperiden, oxybutinin (antiparkinsonian), butylscopalamine (gastrointestinal cramps), scopalamine (for nausea, e.g. motion sickness), dimenhydrinate (for nausea, sedation), antihistamines (for allergies)	Inhibition of central temperature regulation → reduced sweating, cognition impaired; drops in blood pressure but also blood pressure crises
Antihypertensives (AHT, high blood pressure medication)	Beta-blockers, ACE inhibitors, sartans, calcium antagonists, clonidine, moxonidine (the latter centrally active)	On the one hand, prevention of dilation of the blood vessels (beta blocker) in the skin → reduced heat dissipation through convection, increase in sweat secretion threshold (central AHT); on the other hand, enhancement of the antihypertensive effect through vasodilation → drops in blood pressure
Diuretics (as high blood pressure medication or for cardiac insufficiency)	Hydrochlorothiazide, contained in many combination drugs, chlorthalidone, indapamide, torasemide, furosemide, spironolactone	Intensification of blood pressure reduction, dehydration, kidney failure, dangerous electrolyte imbalance
Antianginosa (for coronary heart disease)	Nitrates such as ISMN, ISDN, molsidomine	Particular vasodilatory effect → dangerous drops in blood pressure
Antiepileptic drugs	Carbamazepine, valproic acid, lamotrigine, benzodiazepines, barbiturates	Impairment of cognitive alertness, increase in electrolyte imbalances (hyponatremia) [55, 56]
Painkillers	NSAIDs (ibuprofen, diclofenac, coxibe), opiates/opioids (fentanyl patches, oral medications such as morphine, hydromorphone, codeine, dihydrocodeine, pethidine, tilidine, tramadol)	Risk of renal failure, triggering of hypertension, worsening of cardiac insufficiency in case of exsiccosis, risk of accumulation of opiates/opioids or increased release of active substance in transdermal systems → cognitive impairment, respiratory depression, anticholinergic side effects
Insulins	Basal insulins, rapid-acting insulins, etc.	Under heat conditions they potentially flow more rapidly increasing the risk of hypoglycaemia (low blood sugar)
SGLT2 inhibitors (for diabetes, heart failure, and renal failure)	Dapagliflozin, empagliflozin, ertugliflozin	Risk of exsiccosis, increase in risk of atypical ketoacidosis due to volume deficiency

ACE=angiotensin-converting enzyme, AHT=antihypertensives, COPD=chronic obstructive pulmonary disease, ISDN=isosorbide dinitrate, ISMN=isosorbide mononitrate, NSAIDs=non-steroidal anti-inflammatory drugs, SGLT2 inhibitor=sodium glucose co-transporter-2 inhibitor

**Table 2:** Population groups at greater health risk during heat events Source: Updated and expanded based on World Health Organization [31]

Category	Risk group
Physiological adaptive capacity	► Older persons (>65 years) ► Infants and young children ► Pregnant women
Pre-existing conditions	► Cardiovascular diseases (e.g. arterial hypertension, coronary heart disease, cardiac insufficiency) ► Cerebrovascular diseases (e.g. stroke) ► Respiratory diseases (e.g. COPD, bronchial asthma) ► Metabolic diseases (e.g. diabetes mellitus) ► Neurological diseases (e.g. Parkinson’s disease due to impaired thermoregulation) ► Mental health conditions (e.g. depression, schizophrenia, drug addiction) ► Kidney diseases (e.g. renal insufficiency) ► Obesity ► Those taking certain medicines to treat the diseases mentioned (see Table 1)
People with disabilities	► Physical disabilities (e.g. spinal cord injuries [58]) ► Mental disabilities, as people with severe cognitive impairments are less able to protect themselves from heat
Functional limitations	► Being confined to bed ► Residing in care facility
Socioeconomic factors	► Social isolation, especially in old age ► Homelessness ► Unfavourable housing situation
Physical exertion in high outdoor temperatures	► People working outdoors (e.g. in agriculture, construction) ► Outdoor sports ► Health workers, especially in combination with personal protective equipment
Workers who cannot leave their workplace during heat events despite high indoor temperatures	► Personnel in medical and care facilities, especially in combination with personal protective equipment

COPD=chronic obstructive pulmonary disease

**Table 3:** Recommendations for strengthening heat-related health protection in Germany (As at end of May 2023)

Goal	Measure	Starting point	Actors	Feasibility issues	Field of action
Systematic and nationwide preparation and implementation of HHAPs	Establish a central coordination office, enable, facilitate, and broaden financial support for municipalities by the federal and state governments	Individual municipalities have already drawn up HHAPs and are starting to implement them; there are good-practice examples for the preparation	Federal, state, and municipal administrations	Dependent on municipalities’ human and financial resources; regulatory requirements such as state public health laws and federal legislation	Structural prevention, health promotion, health and civil protection
Strengthening the healthcare sector in its central role in heat-related health protection and the corresponding precautionary measures	Activation of healthcare actors as a central pillar of HHAPs, especially for the implementation of acute preventive adaptation measures	First action alliances in the health sector [84]	Healthcare facilities, nursing homes and care services, general practitioners, public health service, fire brigade and emergency services, associations and federations, volunteers	Sense of medical responsibility; initiative of actors and protagonists	Healthcare
Raising awareness and competence in dealing with heat in the population	Communication strategy	A wide range of information material is already available	Federal, state, and municipal administrations	Coordination of federal, state, and local governments, different channels required	Health awareness, health promotion, health protection
Promptly assess health effects due to heat stress on a small scale and in hospitals and care facilities	Expand realtime monitoring of heat mortality, establish monitoring of morbidity and labour situation in hospitals and care facilities during heat events	Realtime mortality surveillance at the Robert Koch Institute, HEAT projects in Hesse [15]	Municipalities, federal states, federal administration	Effort for collection, analysis, and storage of the data	Surveillance
Heat-resilient health system	Investing in the healthcare system to strengthen the reliability of infrastructures in the event of heat	Existing hospital alert and emergency plan	Federal administration, federal states	Funding opportunities limited so far	Funding and resources
Heat-literate healthcare personnel	Education and further training of healthcare personnel in heat protection/provision, prevention campaigns [71, 77, 78, 85]	Existing training offers and materials, e.g. projects at LMU on heat-health protection in care	Federal administration, federal states	Existing training courses so far do not extensively cover the topic of heat-health protection	Education
Heat-resilient building structure with special attention to hospitals and nursing homes	Review and, if necessary, adapt building and occupational health and safety legislation [71] with regard to their effectiveness for heat-related health protection	Special protection of healthcare personnel required; uphold existing legislation on passive cooling of buildings	Federal administration, federal states	Implementation based on the recommendations of the European Network Architecture for Health [86]	Urban and spatial planning, occupational health and safety
Ongoing, evidence-based implementation and further development of HHAPs	Evaluation of HHAPs and measures	Municipalities with implemented HHAPs; WHO Europe methodology and other regions, e.g. England [87, 88]	Monitoring and analysis of HHAP implementation in federal states and municipalities by the federal administration	Distribution of responsibilities between the environment and health sectors unclear	Structural prevention, health promotion, health and civil protection
Evidence-based treatment of patients with regard to risks associated with heat	Concept development for dealing with heat-sensitive medication, e.g. summary of evidence, feasibility studies, development of an alert app	State of knowledge on interactions of medication and high temperatures	Federal administration, federal states	So far no guidelines/standards for medication administration during heat (dosage adjustment to suspension)	Training, communication

HHAP=Heat-health action plan, LMU=Ludwig-Maximilians-Universität, WHO=World Health Organization
